# Proteomic Analysis of the Early Development of the *Phalaenopsis amabilis* Flower Bud under Low Temperature Induction Using the iTRAQ/MRM Approach

**DOI:** 10.3390/molecules25051244

**Published:** 2020-03-10

**Authors:** Cong Chen, Lanting Zeng, Haiyi Zhao, Qingsheng Ye

**Affiliations:** 1 Guangdong Provincial Key Lab of Biotechnology for Plant Development, School of Life Sciences, South China Normal University, Guangzhou 510631, China; parker_cc@163.com (C.C.); zenglanting@scbg.ac.cn (L.Z.); 2 Lingnan Institute of Technology, Guangzhou 510663, China; 3 Genecreate Biological Engineering Co., Ltd., Wuhan 430075, China; haiyi.zhao@genecreate.com

**Keywords:** *Phalaenopsis amabilis*, low temperature, flower bud, iTRAQ, MRM

## Abstract

*Phalaenopsis amabilis*, one of the most important plants in the international flower market due to its graceful shape and colorful flowers, is an orchid that undergoes vernalization and requires low-temperature treatment for flowering. There have been few reports on the proteomics of the development of flower buds. In this study, isobaric tags for relative and absolute quantification (iTRAQ) were used to identify 5064 differentially expressed proteins in *P. amabilis* under low-temperature treatment; of these, 42 were associated with early floral induction, and 18 were verified by mass spectrometry multi-reaction monitoring (MRM). The data are available via ProteomeXchange under identifier PXD013908. Among the proteins associated with the vernalization pathway, PEQU_11434 (glycine-rich RNA-binding protein GRP1A-like) and PEQU_19304 (FT, VRN3 homolog) were verified by MRM, and some other important proteins related to vernalization and photoperiod pathway that were detected by iTRAQ but not successfully verified by MRM, such as PEQU_11045 (UDP-N-acetylglucosamine diphosphorylase), phytochromes A (PEQU_13449, PEQU_35378), B (PEQU_09249), and C (PEQU_41401). Our data revealed a regulation network of the early development of flower buds in *P. amabilis* under low temperature induction.

## 1. Introduction

The flowering process, including the induction of flowering and flower evocation, of higher plants is the central link of plant development and starts from the initial decision to flower under specific environmental conditions. Vernalization is an example of the influence of temperature on the timing of flowering. The molecular basis of vernalization revealed that vernalization systems evolved independently in different plant groups [1]. However, the distinct vernalization pathway shares a common principle that there is a block to flowering and cold provides competence to flower in vernalization-responsive species [2]. Moreover, the induction of flowering typically requires additional inductive signals, which can occur long after a return to warmer growth temperature [2]. Previous study has proposed that vernalization and the chilling requirement to exit bud dormancy are under shared regulation [3]. With the completion of whole-genome sequencing for model plants and continuous exploration of the mechanism of plant floral induction, four floral induction pathways for Arabidopsis thaliana, namely, the vernalization pathway, the light-dependent pathways, the autonomous pathway, and the gibberellin pathway have been revealed [4]. At present, most studies have focused on dicotyledonous model plant *Arabidopsis thaliana* [5] and monocotyledonous crop wheat [6] of the Gramineae family, whereas there have been very few studies on Orchidaceae family monocotyledons such as *Phalaenopsis amabilis* [7].

*Phalaenopsis* species are produced in large quantities annually and are marketed as the most important potted plants worldwide because of their beautiful appearance [8]. *P. amabilis* is a vernalization-responsive species that requires vernalization during the vegetative growth stage and appropriate photoperiodic treatment before flowering occurs [9]. In native cultivation locations, the natural flowering period of *P. amabilis* is from March to May. Artificial regulation of the flowering period of *P. amabilis*, blossoming in time for the Spring festival and other holidays, is inevitable for increasing its commodity value. A low temperature is required for *P. amabilis* flowering. Previous studies on the regulation of flowering in *P. amabilis* have focused on physiological and molecular aspects [10,11], although there have been no reports of proteomic research on floral development of *P. amabilis*. At the same time, the key genes and regulatory mechanism of the flowering process in *P. amabilis* are not clear. In this study, morphological and histological observations of flower bud differentiation were performed to have a comprehensive phenotypic profile of bud differentiation in *P. amabilis*. In addition, we also determined differential protein expression in the developing *P. amabilis* floral bud under low-temperature treatment to screen out the candidate genes regulating the bud differentiation in *P. amabilis*. The analysis of proteomics changes in *P. amabilis* flower buds combined with phenotypic analysis under low temperature induction are helpful for understanding the mechanism of flowering in *P. amabilis* and better controlling flowering time, at least providing some basic data for gene level control of the flowering time of *P. amabilis*.

## 2. Results

### 2.1. Morphological Observation of Flower Bud Differentiation

**Figure 1:**
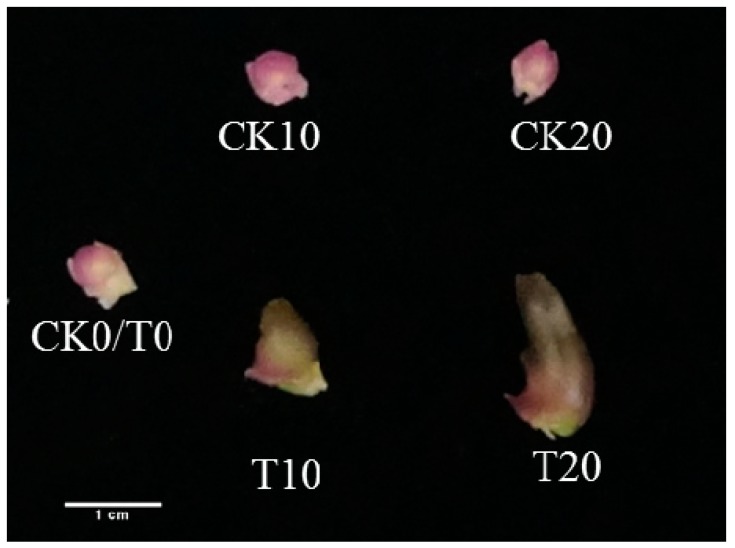
Morphological changes of *P. amabilis* flower buds at different stages in control conditions and two treatments. CK0, CK10, and CK20 represent the control group grown under normal temperatures for 0 d, 10 d, and 20 d, respectively; T0, T10, and T20 represent the treatment group after low temperature induction for 0 d, 10 d, and 20 d, respectively.

After low temperature induction for 10 d, the transition from a vegetative to a reproductive stage began in *P. amabilis* flower buds. The buds hidden in the third and fourth leaf axils (from the top of the shoot) began to expand and grow; then, they broke through and emerged from the leaf axils before continuing to extend. During the process of low temperature induction, buds at different stages were cut from the control condition (CK) and treatment group plants. The morphological observations revealed that significant changes occurred in *P. amabilis* flower buds under low-temperature treatment; before low temperature induction, there were already buds inside the third and fourth leaf axils (counted from the top of the shoot) with pink tips and yellow bases. After 10 d of low temperature induction, the buds expanded, and their color deepened; after 20 d, the buds grew quickly and developed a dark green color on their tips. However, in the control group (induced under normal temperature), no morphological change was observed in the flower buds (Figure 1).

### 2.2. Histological Observation of Flower Bud Differentiation

**Figure 2:**
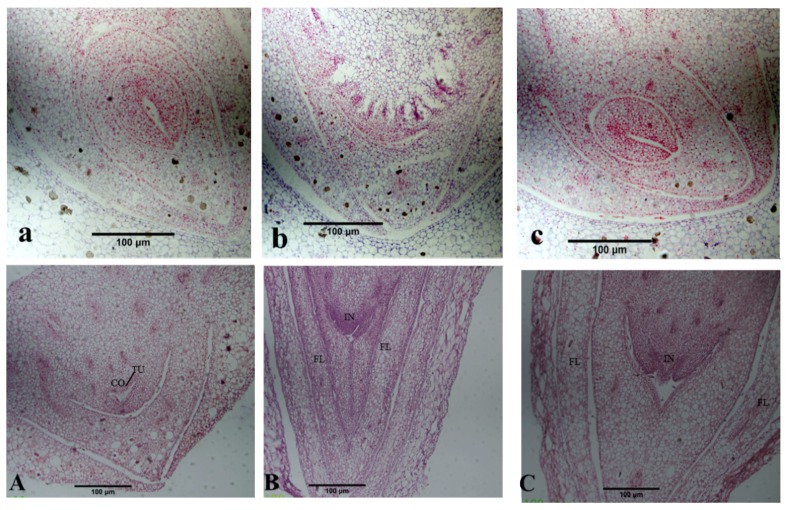
Micro-structural change of *P. amabilis* flower buds at different treatment stages. (**a**–**c**) represent the control group grown under normal temperatures for 0 d, 10 d, and 20 d, respectively; (**A**–**C**) represent the treatment group after low temperature induction for 0 d, 10 d, and 20 d, respectively. TU, tunica; CO, corpus; GP, growth cone; IN, inflorescence primordium; FL, floral primordia.

As shown in the microstructure of paraffin-embedded *P. amabilis* flower bud sections (Figure 2), before low temperature induction, the buds contained a growth cone with a typical tunica and corpus zones. The tunica zone comprised one or several layers of cells on the surface of the growth cone that were smaller in size and had thicker cytoplasm than cells from the corpus zone and were tightly arranged. The nuclei of the cells in this zone were larger and more darkly stained than those in the corpus zone. The corpus comprised cells in the central zone that were larger in size and had thinner cytoplasm than cells in the tunica zone. They were loosely arranged, and the nuclei of the cells in this zone were smaller and more lightly stained than were those in the tunica zone. After low temperature induction for 10 d, a transition from vegetative growth to reproductive growth was observed that enlarged the protruding growth cone and formed the inflorescence primordium and floral primordial tissue. After low temperature induction for 20 d, the enlarged growth cone protruded in the base and differentiated to form oval protrusions. However, the flower buds in the control plants remained in a state of dormancy.

### 2.3. Quantitative Identification of Proteins by Isobaric Tags for Relative and Absolute Quantification (iTRAQ)

**Figure 3:**
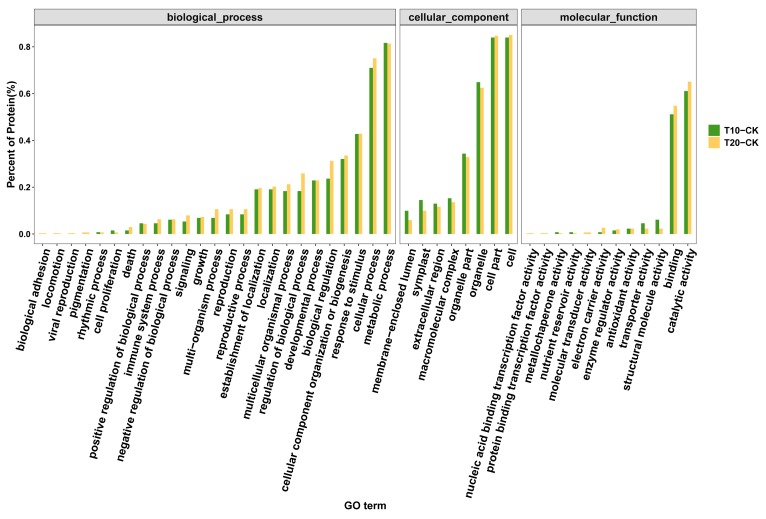
Gene Ontology (GO) functional classification of significantly different expressed proteins identified by isobaric tags for relative and absolute quantification (iTRAQ). The distribution of GO functional classifications involved in the two treatments. Different GO functional classifications for genes involved in the two treatments are marked with different colors. The length of the bar represents the number of different expressed proteins annotated in each class.

**Figure 4:**
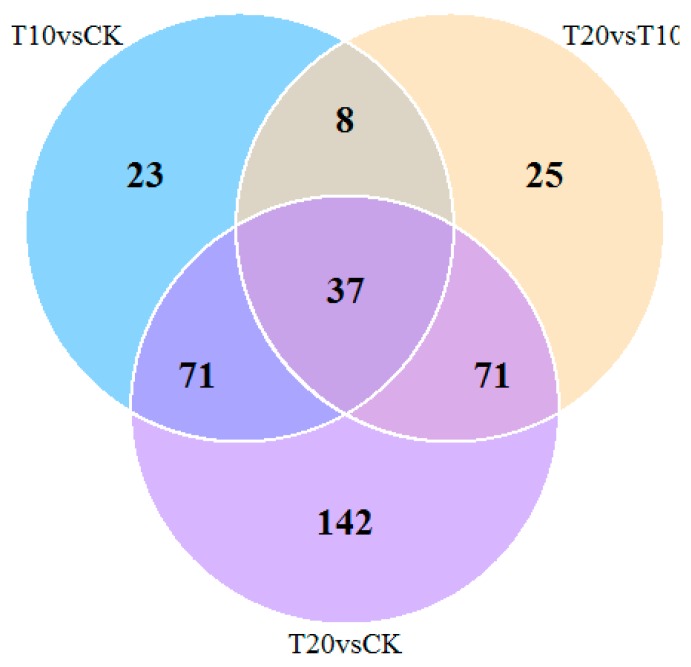
Venn diagram of differentially expressed proteins. The number of proteins identified to be significantly changed in the control and treatment samples were 139 (T10 vs. CK), 321 (T20 vs. CK), and 141 (T20 vs. T10), of which the regulation intensity was more than 1.5 times or less than 0.67 times (*p* ≤ 0.05).

**Figure 5:**
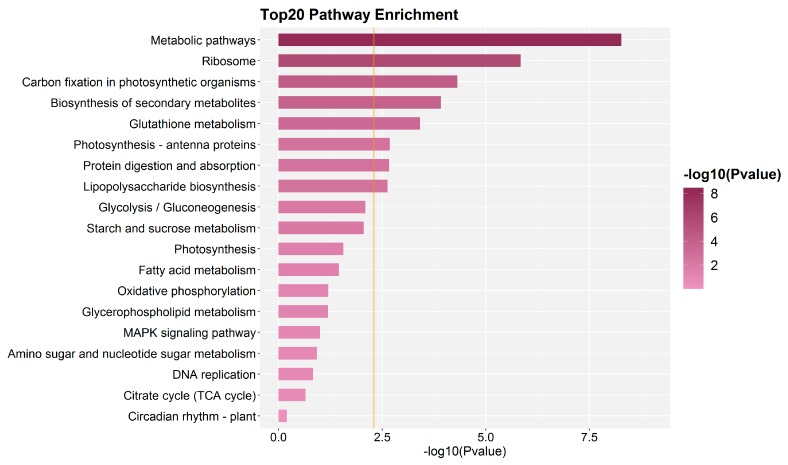
Top ranked pathways that are significantly altered in treatment groups as compared with CK. The x-axis represents log10 (*p*-value).

Using the isobaric tags for relative and absolute quantification (iTRAQ) approach, 5064 proteins were identified and annotated (including Gene Ontology (GO) and pathway analysis) during floral bud induction in *P. amabilis*. A total of 4960 proteins overlapped between the control and treatment groups. The GO functional classification of significantly different expressed proteins is shown in Figure 3. As shown in Figure 4, the number of proteins identified to be significantly changed in control and treatment samples were 139 (T10 vs. CK), 321 (T20 vs. CK), and 141 (T20 vs. T10), of which the regulation intensity was more than 1.5 times or less than 0.67 times (*p* ≤ 0.05). The top ranked pathways that are significantly altered in treatment groups as compared with CK are shown in Figure 5. On the basis of the metabolic profile related to flower development in the vernalization pathway, light-dependent pathways, autonomous pathway, and gibberellin pathway, 42 proteins related to photoperiod, the vernalization pathway, the hormone pathway, carbon metabolism, energy metabolism, and the stress response were identified. The fold changes in expression levels of these proteins are shown in Table 1.

### 2.4. MRM Verification

We, then, performed MRM verification of the 42 proteins identified by iTRAQ, 18 of which were verified (Table 2). The observed trends in their expression levels were roughly consistent with the results obtained by iTRAQ. However, no phytochrome-related proteins or acetyl-glucosamine diphoshorylases were identified by MRM. There are a variety of reasons why the data were not identified by MRM, as follows: protein homology was high; unique peptides were not identified; protein overly abundant peptide segments; low sensitivity of the instrument; some ions in the sample were similar to the target protein transition; and a high level of interference, which is not suitable for quantification. Therefore, it is impossible to successfully build methods for all target proteins. Among the verified proteins, PEQU_11434 (glycine-rich RNA-binding protein GRP1A-like) was significantly upregulated during low-temperature treatment as compared with that observed in control flower buds, suggesting that upregulated expression of GRP induces the development of floral buds at low temperature. This is consistent with the results from the iTRAQ experiment. The ratios of PEQU_19304 (FT) expression in groups T10/CK, T20/CK, and T20/T10 were 2.44, 2.59, and 1.06, respectively, which indicated significantly upregulated expression of the FT protein with cold treatment as compared with that observed in control flower buds. This is consistent with the iTRAQ results.

## 3. Discussion

### 3.1. Photoperiod-Related Proteins

In the model plant *Arabidopsis thaliana*, a change in the expression pattern of CO (Constans)/FT genes is the hallmark of the photoperiodic induction pathway [12]. CO is a typical timing control gene that encodes one photostable protein containing two zinc finger motifs (B-box) and that regulates interaction between proteins through its N-terminal domain. The C-terminal end contains a CCT (CO, CO-like, timing of CAB expression 1) domain that is required for nuclear localization [13]. CO is a transcription factor without a distinct DNA-binding domain. It interacts with the FT promoter domain and is the main positive regulator of FT [14]. In this study, we identified two zinc finger family proteins using the iTRAQ approach, namely, PEQU_02392 (zinc finger CCCH domain-containing protein 4-like, FD) and PEQU_11519 (LON peptidase N-terminal domain and RING finger protein 1). The relationship between these two proteins and CO requires further research.

In addition to photoperiod, light quality affects the stability of the CO/FT expression pattern. Under blue light regulation, GI can bind to two other ubiquitin proteins, ZTL (Zeitlupe) and LKP2 (LOV Kelch repeat protein 2), to degrade the CDF (Cation diffusion facilitator proteins) 2,3,5 and jointly regulate flowering in *A. thaliana* [15,16]. The stability of the CO protein is positively regulated by the blue light receptor cryptochromes CRY1 and CRY2 and red/far red light receptor phytochrome A (phyA) and negatively regulated by phyB [15], the E3 ubiquitin ligase protein COP1 (CONSTITUTIVE PHOTOMORPHOGENESIS 1) [17] and FKF1 (FLAVIN-BINDING, KELCH REPEAT, F-BOX 1) [18]. In this work, CRY1, phyA, phyB, and phyC were downregulated as compared with their expression in control flower buds, suggesting that the regulation of light quality in the monocot *P. amabilis* could be different from that in *A. thaliana*. In this study, FHA domain-containing protein DDL and bibenzyl synthase were upregulated and allene oxide synthase 2-like; oxygen-evolving enhancer protein 2, chloroplastic-like; and oxygen-evolving enhancer protein 1, chloroplastic-like were significantly upregulated as compared with their expression in control flower buds, suggesting that these genes are related to photosynthesis.

### 3.2. Vernalization-Related Proteins

Many RNA-binding proteins have RNA recognition motif (RRM) or K homology (KH) domains related to RNA-binding activity. The KH module of KH domain-containing proteins is a widespread RNA-binding motif utilized by heterogeneous nuclear ribonucleoprotein K (hnRNP K) and ribosomal protein sequences. Previous studies have shown that FLOWERING LOCUS WITH KH DOMAINS (FLK) and FLOWERING LOCUS PA (FPA), two KH domain-containing proteins in *A. thaliana*, process the mRNA of the flowering inhibitor FLOWERING LOCUS C (FLC) [19]. In recent years, the FLC transcriptional factor in the MADS-box gene family has been a hot topic in vernalization research. Studies by Rodríguez-Cazorla et al. [20] further indicated that FLK (FLOWERING LOCUS WITH KH DOMAINS) and PEP (PEPPER) interact to regulate FLC (FLOWERING LOCUS C), the central repressor of flowering time. FLK and PEP regulate the function of the C-terminus of the MADS-box flower homology gene AGAMOUS (AG) to maintain flora organ recognition through posttranscriptional regulation. In this experiment, both GRP1A-like glycine-rich proteins and KH domain-containing proteins were upregulated with cold treatment as compared with their expression in control flower buds, indicating that KH domain-containing proteins play a vital role in plant flowering.

Approximately 2% to 5% of intracellular glucose enters the hexosamine metabolism pathway and eventually generates UDP-GlcNAc [21]. In this work, PEQU_11045 (UDP-N-acetylglucosamine diphosphorylase 1-like) was found to be upregulated with cold treatment as compared with its expression in control flower buds and catalyzed the transformation of GlcNAc-1P to UDP-G1cNAc. Xiao et al. [6] revealed a novel mechanism for controlling *TaVRN1* mRNA accumulation in response to prolonged cold sensing in wheat. The carbohydrate-binding protein VER2, a jacalin lectin, promotes TaVRN1 upregulation by physically interacting with the RNA-binding protein TaGRP2. TaGRP2 in turn binds to TaVRN1 pre-mRNA to inhibit accumulation of TaVRN1 mRNA. The physical interaction between VER2 and TaGRP2 is controlled by TaGRP2 O-GlcNAc modification, which gradually increases during vernalization. The interaction between VER2 and O-GlcNAc-TaGRP2 reduces TaGRP2 protein accumulation in the nucleus or promotes TaGRP2 dissociation from TaVRN1, leading to TaVRN1 mRNA accumulation.

### 3.3. Ubiquitin-Related Proteins

In *Arabidopsis*, GA (gibberellin) regulates flowering by binding to GID1 (gibberellin-insensitive dwarf 1) to stimulate formation of the GA-GID1-DELLA complex [22]. Based on labeling by the SCF (SKP1-CUL1-F-box) polymer, it was found that after the formation of GA-GID1-DELLA, DELLA was degraded by the ubiquitin 26S proteasome, thus, reducing DELLA interaction with SPL9 (SQUAMOSA-promoter binding-like protein 9), which is beneficial for expression of downstream LFY and SOC1 [22,23,24]. The GA-GID1-DELLA signaling pathway with GA transduction is mediated by the ubiquitin proteasome degradation pathway to achieve flowering induction [23,24].

Ubiquitin degradation pathways include ubiquitin-activating enzyme E1, ubiquitin ligase E2, ubiquitin ligase E3, and the 26S proteasome. CUL1 is a ubiquitin ligase E3 that is expressed throughout the entire life cycle of *A. thaliana*, from zygophase to pollen formation [25]. In this work, five ubiquitin ligases were found to be differentially expressed under cold treatment, some of which were upregulated and some downregulated. The mechanism of this differential expression requires further research.

### 3.4. Other Proteins

Proteins related to the stress response, for example, catalase isozyme A, carbon metabolism (e.g., glyceraldehyde-3-phosphate dehydrogenase A), energy metabolism (e.g., ferredoxin-NADP reductase), amino acids (e.g., S-adenosylmethionine synthase), protein metabolism (e.g., protein IN2-1 homolog B), and posttranscriptional modification (e.g., methyltransferase) account for more than half of the total proteins that were identified as being differentially expressed. This partly demonstrates the importance of related metabolic activities in the development of flower buds. In this study, some stress response-related proteins, such as beta-glucosidase 1-like and alcohol dehydrogenase class-3, were downregulated, whereas others, such as GDP-mannose 3,5-epimerase 2 and catalase isozyme A, were upregulated. This could be due to the gradual adaptation of flower buds to low-temperature conditions over time. Moreover, posttranscriptional modification increased, and posttranscriptional modification-related proteins such as probable methyltransferase PMT2 tended to be upregulated as compared with expression in control flower buds. Because the development of flower buds is closely related to carbon and energy, metabolism in cold-treated flower buds increased compared to that observed in control flower buds [26]. Additionally, to meet the need for amino acids and proteins during flower bud development, amino acid and protein metabolism both increased as compared with that observed in control flower buds.

### 3.5. Possible Mechanism for the Regulation of the Early Development of Flower Bud in P. amabilis under Low Temperature 

**Figure 6:**
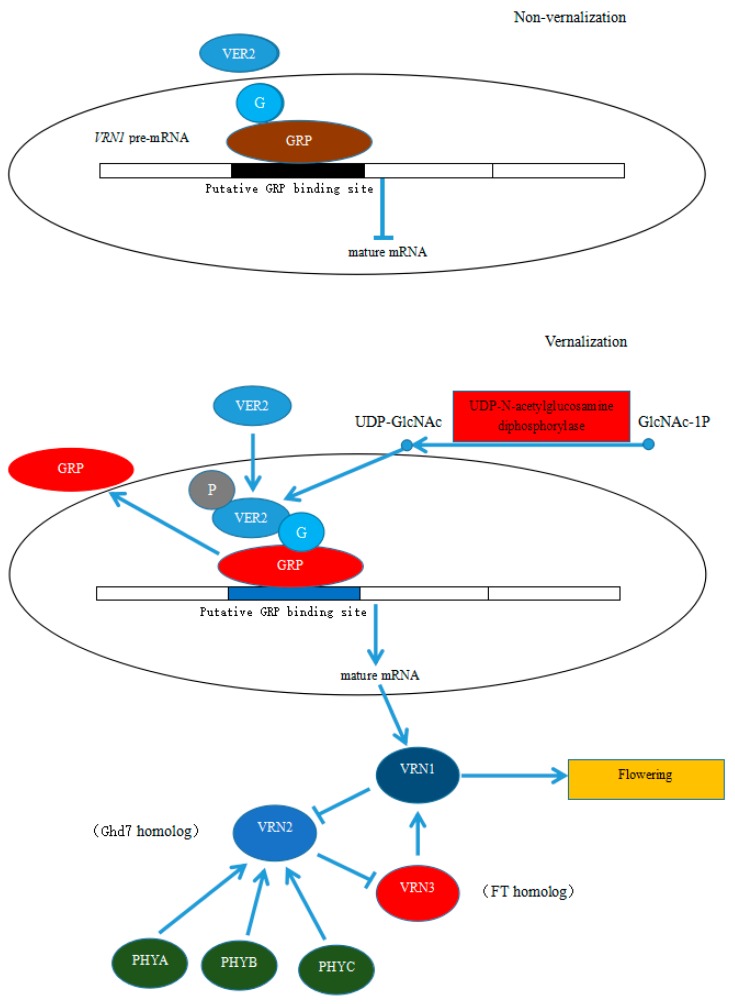
Putative regulatory network of early flower bud differentiation in *P. Amabilis* induced at low temperature. Red ovals represent the upregulated expression of a protein, and green ovals represent the downregulated expression of a protein. Diagram is based on Xiao et al. [6] with some modifications.

Many plants grown in temperate climates require exposure to prolonged low temperature in winter to initiate the flowering process in spring, a process called vernalization [27]. In model plants such as *A. thaliana* and cereals such as wheat and barley, a variety of genes that regulate flowering under vernalization conditions have been identified [28,29,30,31,32,33,34]. The key gene that regulates flowering in the dicotyledon *A. thaliana* is *FLC* (*FLOWERING LOCUS C*), and in the monocots wheat and barley is *VRN1*. The protein encoded by the FLC gene in *A. thaliana* is a strong inhibitor of flowering, and high levels of expression suppress flowering [31,32,33,34]. The level of *TaVRN1* transcription in cereals increases dramatically after vernalization as compared with that observed before vernalization, and high expression levels of *VRN1* promote flowering [32,34,35]. The flowering-related genes identified by Liang et al. [36] during the flowering transition process in *Dendrobium nobile* Lindl under low temperature induction included genes homologous to the key gene in the vernalization pathway, such as *VRN1* and *FT/VRN3* in cereal plants and *AGL19* in *A. thaliana*. The regulatory network of these genes in *D. nobile* Lindl is consistent with that in cereal plants and *A. thaliana*. *FLC* is the key gene in the vernalization pathway in *A. thaliana*, although no related homologous gene has been cloned in monocotyledonous plants. Thus far, the *VRN1* pathway is the only vernalization pathway that has been identified in monocotyledonous plants. *VRN1* is homologous to *AP1/FUL* in *A. thaliana,* and its expression is upregulated after vernalization [37]. *P. amabilis* belongs to the Orchidaceae family of the Monocotyledoneae class. We inferred that its flowering pathway under low temperature induction would be the same as that of cereal plants in Monocotyledoneae, with *VRN1* as the key gene. PEQU_11434 (glycine-rich RNA-binding protein GRP1A-like), the only glycine-rich RNA-binding protein identified by our iTRAQ analysis, was upregulated as compared with its expression in control flower buds, consistent with the results of MRM verification. Wheat TaGRP2, a member of the GR-RBP (GRP) family [38], could directly regulate TaVRN1 transcript accumulation [6]. In *Arabidopsis*, the ortholog AtGRP7 is proposed to regulate the mRNA processing of genes involved in flowering and stress response by directly binding to their transcripts [39]. Furthermore, expression of PEQU_11045 (UDP-N-acetylglucosamine diphosphorylase 1-like) was upregulated as compared with its expression in control flower buds. Glucose generates UDP-GlcNAc through the hexosamine biosynthesis pathway. Under low temperature induction, the level of UDP-GlcNAc was increased as compared with that observed in control flower buds, leading to an increase in TaGRP2O-GlcNA glycosylation [6]. VER2 reacts with O-GlcNAc-glycosylated TaGRP2 and promotes its dissociation from *TaVRN1* pre-mRNA to enable expression of *TaVRN1*. *VRN1* is a homolog of *AP1/FUL* in *A. thaliana*, and its high-level expression promotes flowering (as shown in Figure 6) [40].

*VRN2* encodes a CCT (Constans, constans-like, timing of CAB expression 1) protein and has no known homolog in *A. thaliana*. It positively regulates flowering under long-day (non-vernalization) conditions and blocks the flowering of wheat in summer and autumn [41]. The homolog of *VRN2* in the short-day plant rice is *Ghd7*, which also suppresses flowering under long-day conditions [42]. Although we did not identify CCT proteins and proteins homologous to Ghd7 in this work, phyA alone or in combination with phyB and phyC can induce accumulation of *Ghd7* mRNA, although phyB alone can decrease the level of *Ghd7* mRNA to a certain degree. In addition, phyB and phyA can affect the activities of Ghd7 and Hd1, respectively [43]. Studies by Strasser et al. [44] indicated that photosensitive pigment-mediated light signals can be integrated into the vernalization pathway and participate in the regulation of *FLC* transcription. VASCULAR PLANT ONE–ZINC FINGER1 (VOZ1) and VOZ2, as phyB-interacting factors, repress flowering by indirectly regulating expression of FT. The *voz1 voz2* double mutants, but neither single mutant, shows a late-flowering phenotype under long-day conditions, which indicates that VOZ1 and VOZ2 redundantly promote flowering. In voz1 voz2 mutants, the early-flowering phenotype of the phyB mutant is suppressed, and FT expression is repressed [45,46]. In this study, we identified two phyA proteins (PEQU_13449 and PEQU_35378), one phyB protein (PEQU_09249), and one phyC protein (PEQU_41401) by iTRAQ, all of which were downregulated as compared with their expression in control flower buds, suggesting that phytochromes A, B, and C are downregulated under low temperature induction to repress expression of the *VRN2* gene, and thus release *VRN3* from *VRN2* repression to enable high expression of FT. Yan et al. [47] found that the vernalization gene *VRN3* in wheat and barley is a homolog of the *FT* gene, playing a role in transmitting vernalization signals that is similar to its role in *A. thaliana*. Expression of PEQU_19304 (FT), which was identified by iTRAQ and verified by MRM, in this work was upregulated as compared with its expression in control flower buds. In summary, under long-day (non-vernalization) conditions, expression of high levels of *VRN2* represses *VRN3*; after vernalization, induced expression of *VRN1* releases *VRN3* from *VRN2*-mediated repression, and the increased levels of *VRN3 (FT)* stimulate mass expression of *VRN1* and promote flowering by interacting with *FDL2* (flowering locus-like 2) protein [41,47,48] (Figure 6).

The implications of the co-isolation problem in iTRAQ do exist in iTRAQ quantification and have been discussed in many methodological literatures [49,50]. Although there is an effect on ratio compression, it does not affect the application of iTRAQ in quantitative proteomics. In addition, to confirm the reliability of iTRAQ quantitative data, the important potential biomarker can be further validated by MRM or Western blot analyses. In future studies, we will explore its molecular mechanisms using validating experiments.

## 4. Materials and Methods 

### 4.1. Plant Materials

*P. amabilis* “Dtps. Ney Shan Gu Niang” was purchased from the floriculture base of the Guangdong Academy of Agricultural Sciences. Five hundred pots of healthy, mature, disease-free and insect-free *P. amabilis* seedlings with a consistent growth status above the 4-leaf stage were selected in 2015 and placed in an artificial growth climate chamber (intensity 250 ± 30 μE·m^−2^·s^−1^) for the low-temperature treatment. Treatment was performed at the temperatures suggested by Huang et al. [51], which were 25 ± 1 °C/20 ± 1 °C (day/night) for the treatment group and 30 ± 1 °C/25 ± 1°C for the CK (control check) group for 12 h/12 h (day/night) each day under plastic greenhouse (shading rate 50%). Flower buds were taken from the axil of the 4th functional leaf (from the top of the shoot) of *P. amabilis* treated for 0 d, 10 d, and 20 d at normal temperature and at low temperature. The flower buds were frozen in liquid nitrogen and stored at −80 °C for proteome analysis. During the treatment, the seedlings were cultivated under normal water and fertilizer conditions, and Hoagland culture solution was applied once a week [52]. The treatment groups were as follows: CK group (mixed flower buds cultivated at normal temperature for 0 d, 10 d, and 20 d), T10 group (flower buds under low-temperature treatment for 10 d), and T20 group (flower buds under low-temperature treatment for 20 d).

### 4.2. Histological Observation of Flower Bud Development

Paraffin sectioning is one of the most commonly used methods for the observation of plant tissue. Paraffin sectioning was conducted as recommended by Sun et al. [53] with the following modifications. The flower buds were fixed with FAA fixation liquid (10% (*v*/*v*) formaldehyde, 50% (*v*/*v*) absolute ethanol, and 5% (*v*/*v*) acetic acid), dehydrated through a graded ethanol series and embedded in paraffin (Paraplast Plus; Sigma Chemical Co., St Louis, MO, USA). Serial sections 5 to 20 µm thick were obtained with a microtome (RM 2255; Leica, Wetzlar, Germany). The sections were dewaxed in xylene, rehydrated through a graded ethanol series, and stained with 2% safranin dissolved in 50% ethanol. Sections 5 to 20 μm thick were embedded using a standard procedure, dewaxed with 50% ethanol and stained with Ehrlich’s hematoxylin for 1 to 2 d. Any residual color was removed with 95% alcohol, and the sections were hyalinized with dimethylbenzene; the slides were sealed with neutral gum. The sections were viewed under a fluorescence inverted microscope (Leica, Wetzlar, Germany).

### 4.3. Protein Extraction

Protein extraction was performed according to a previously described method [54], with some modifications. Flower buds (0.5 g) were ground to powder in liquid nitrogen and incubated in dissolution buffer (8 M urea, 100 mM tetraethylammonium bromide (TEAB), pH 8.0) on ice. The mixture was centrifuged for 20 min at 13,000× *g*, and the collected supernatant was precipitated using precooled acetone containing 10 mM dithiothreitol (DTT) at −20 °C for 2 h. After centrifugation at 13,000× *g* for 20 min at 4 °C, the precipitate was collected, and 800 μL of chilled acetone (containing DTT at a final concentration of 10 mM) was added. The sample was centrifuged at 13,000× *g* for 20 min at 4 °C. The precipitate was collected, air-dried, and dissolved in 100 µL dissolution. The protein content was determined by the Bradford method [55].

For each sample, 80 µg of protein was dissolved in 100 µL of dissolution buffer and diluted in 500 µL of 50 mM ammonium bicarbonate (NH_4_HCO_3_). Protein samples were reduced with 10 mM DTT at 56 °C for 30 min and alkylated with 50 mM iodoacetamide (IAM) at room temperature for 30 min in the dark. Two micrograms of trypsin were added and incubated overnight at 37 °C for protein digestion; an equal volume of 0.1% formic acid (FA) was, then added to acidify the peptides. The peptides were purified using a Strata X C18 column (Phenomenex, Torrance, CA, USA) that was first activated with methanol, equilibrated by adding 1 mL of 0.1% FA three times, washed with 0.1% FA + 5% acetonitrile (ACN) twice, and eluted with 1 mL 0.1% FA + 80% ACN. The eluted peptides were dried in a vacuum concentrator. The dried peptide powder was redissolved with 20 µl 0.5 M TEAB for peptide labeling.

### 4.4. iTRAQ Labeling and LC-ESI MS/MS Analysis

The samples were labeled with the following iTRAQ 8-plex kits (AB Sciex UK Limited, Warrington, Cheshire, UK): kits 113 and 114 for CK-1 and CK-2 (biological replicate of CK-1), kits 115 and 116 for T10-1 and T10-2 (biological replicate of T10-1) under low-temperature treatment for 10 d, and kits 117 and 118 for T20-1 and T20-2 (biological replicate of T20-1) under low-temperature treatment for 20 d. The labeled samples were mixed in equivalent amounts and fractionated using an HPLC system (Thermo DIONEX Ultimate 3000 BioRS, Thermo Fisher Scientific, Waltham, MA, USA) with a Durashell C18 column (Welch Materials, Shanghai, China, 5 µm, 100 Å, 4.6 × 250 mm). Thirteen fractions were collected.

The fractionated samples were analyzed by LC-ESI-MS/MS, which was performed using an AB SCIEX nanoLC-MS/MS (AB Sciex, Foster City, CA, USA) system. The samples were chromatographed with a gradient from 2% to 30% (buffer A 0.1% (*v*/*v*) FA, 5% (*v*/*v*) ACN; buffer B 0.1% (*v*/*v*) FA, 95% (*v*/*v*) ACN) for 90 min after direct injection onto a 20 µm PicoFrit emitter (New Objective; packed to 10 cm with Magic C18 AQ 3 µm 120 Å stationary phase). MS1 spectra were collected in the 350e1500 *m*/*z* range for 250 ms. The 40 precursors that were most intense which exceeded 150 counts per second with a 2+ to 5+ charge state were selected for fragmentation, and MS2 spectra were collected in the 50 to 2000 *m/z* range for 100 ms. The dynamic exclusion was set as 12 s. The normalization method was set as the median, and the data were normalized by bias and background corrections. The mass spectrometry proteomics data have been deposited in ProteomeXchange Consortium via the PRIDE partner repository under the dataset identifier PXD013908.

### 4.5. Protein Identification and Proteome Analysis 

MS/MS data were searched using Protein Pilot software v4.5 (AB Sciex, Framingham, MA, USA) against the “*P. amabilis*” subset of the NCBI nonredundant sequence (NR) databases (42294 entries, updated in October 2014) [56]. The parameters were set as follows: iTRAQ 8-plex peptide label, trypsin enzyme, cysteine modified with iodoacetamide, thorough search mode, and biological modifications selected as the ID focus. The false discovery rate (FDR) was estimated using the proteomics system performance evaluation pipeline (PSPEP) algorithm integrated into ProteinPilot, and only proteins with at least one unique peptide and an unused value more than 1.3 were considered for subsequent analysis (equal to protein confidence being set at 95%). For protein quantification, a protein ratio was automatically calculated with ProteinPilot software using the weighted average of log-transformed peptide ratios. Shared peptides and missing values were discarded and not taken into account for protein quantification. The normalization method was set to median, and the data were normalized by bias and background corrections.

### 4.6. MRM Analyses

The samples were digested as described and spiked with 50 fmol of β-galactosidase for data normalization [57,58,59]. MRM analyses were performed using a QTRAP 5500 mass spectrometer (SCIEX, Framingham, MA, USA) equipped with an LC-20AD nano-HPLC system (Shimadzu, Kyoto, Japan). The mobile phase consisted of solvent A (0.1% aqueous formic acid) and solvent B (98% acetonitrile with 0.1% formic acid). The peptides were separated on a C18 column (0.075 × 150 mm column, 3.6 μm) at 300 nL/min and eluted with a gradient of 5% to 30% solvent B for 38 min, 30% ot 80% solvent B for 4 min, and 80% solvent B for 8 min. A spray voltage of 2400 V, a nebulizer gas pressure of 23 psi, and a dwell time of 10 ms were used with the QTRAP 5500 mass spectrometer. Multiple MRM transitions were monitored using unit resolution in both the Q1 and Q3 quadrupoles to maximize specificity.

### 4.7. Data Analysis

We used Skyline [60] software to integrate the raw data generated by the QTRAP 5500 (SCIEX, Framingham, MA, USA). An iRT strategy [61] was employed to define the chromatographic signal from a given peptide against a spectral library. All transitions for each peptide were used for quantitation unless interference from the matrix was observed. Spiked β-galactosidase was used for label-free data normalization. We use MSstats with the linear mixed-effects model, and the P values were adjusted to control the FDR at a cutoff of 0.05. All proteins with a P value below 0.05 and a fold change larger than 1.5 were considered significant.

### 4.8. Functional Analysis of Proteins

Bioinformatics data describing the functional properties of the identified proteins were obtained by GO and KEGG annotations. GO (available online: http://www.geneontology.org) and KEGG pathway (available online: http://www.genome.jp/kegg/pathway.html) analyses were performed with the PartiGene program (available online: http://www.nematodes.org/bioinformatics/annot8r/index.shtml). The annot8r program was used to assign KEGG (gene) pathways and GO (protein) terms based on BLASTX similarity (E-value < 1.0 × 10−5) and known GO annotations.

## 5. Conclusions

In this study, 5064 differentially expressed proteins in flower buds under low-temperature treatment were identified by proteomic analysis; of these proteins, 42 are associated with the photoperiod, vernalization pathway, hormone pathway, carbon metabolism, energy metabolism, and the stress response. PEQU_11434 (glycine-rich RNA-binding protein GRP1A-like) and PEQU_11045 (UDP-N-acetylglucosamine diphosphorylase 1-like), which are associated with vernalization pathways, were upregulated as compared with in control flower buds, inferring that O-GlcNAc glycosylation is involved in the posttranscriptional modification of *VRN1* (API homolog) and that the GRP2 protein (glycine-rich RNA binding protein) is glycosylated to relieve its binding to VRN1 precursor mRNA to promote expression of *VRN1* and flowering. Expression of PEQU_19304 (FT) was upregulated as compared with in control flower buds. After vernalization, the increased expression of *VRN1* released *VRN3* from *VRN2*-mediated repression, and the increased levels of *VRN3* (*FT*) stimulated a high level of *VRN1* expression and promoted flowering by interacting with the FDL2 (flowering locus-like 2) protein. The regulatory mechanism for the early development of *P. amabilis* flower buds is a complicated and coordinated multipathway process. The network proposed above is conjecture based on proteomic results. The specific mechanism of protein coordination requires further empirical verification and research.

## Figures and Tables

**Table 1:** List of upregulated or downregulated proteins related to flower development under low-temperature treatment.

Accession Code	GI Name	% Cov (95)	PI	MW (kDa)	# of Peptides	Fold Change		
						T10/CK	T20/CK	T20/T10
**Photoperiod-Related Proteins**								
PEQU_11519	LON peptidase N-terminal domain and RING finger protein 1	21.560	6.9	39.96	2	1.146 ± 0.127	1.141 ± 0.119	0.986 ± 0.006
PEQU_27404	Protein BTR1	23.560	5.72	26.84	12	1.295 ± 0.067	1.654 ± 0.258	1.275 ± 0.149
PEQU_18278	Chromatin-remodeling protein EBS-like isoform X1	25.560	7.9	24.72	4	0.428 ± 0.053	0.316 ± 0.016	0.696 ± 0.059
PEQU_06861	PHD finger protein ALFIN-LIKE 9-like	26.560	5.64	30.18	3	0.936 ± 0.083	0.875 ± 0.003	1.034 ± 0.061
PEQU_02392	Zinc finger CCCH domain-containing protein 4-like	34.560	6.87	113.85	2	0.838 ± 0.093	0.742 ± 0.106	0.875 ± 0.028
PEQU_16431	PHD finger protein ALFIN-LIKE 6-like isoform X1	35.560	4.75	28.43	3	0.963 ± 0.419	0.723 ± 0.587	0.662 ± 0.319
PEQU_03363	Probable ADP-ribosylation factor GTPase-activating protein AGD9	36.560	8.03	44.08	9	1.035 ± 0.087	1.000 ± 0.013	0.958 ± 0.100
PEQU_09548	Cryptochrome-1-like	12.560	5.1	71.84	6	0.836 ± 0.016	0.825 ± 0.043	0.977 ± 0.032
PEQU_22307	Cryptochrome-1 isoform X1	13.560	5.47	73.66	14	0.823 ± 0.505	0.473 ± 0.101	0.644 ± 0.265
PEQU_41401	Phytochrome C isoform X2	14.560	5.73	125.83	11	0.777 ± 0.277	0.529 ± 0.065	0.688 ± 0.151
PEQU_13449	Phytochrome A	15.560	6.26	125.84	7	0.694 ± 0.121	0.904 ± 0.024	1.305 ± 0.237
PEQU_09249	Phytochrome B isoform X1	16.560	5.4	98.54	5	1.068 ± 0.221	0.802 ± 0.042	0.752 ± 0.112
PEQU_06485	Bibenzyl synthase	27.560	6.43	42.59	4	1.017 ± 0.742	1.927 ± 0.175	2.399 ± 1.511
PEQU_07192	Cryptochrome-1	32.560	5.06	77.8	7	1.067 ± 0.000	1.008 ± 0.124	0.937 ± 0.116
PEQU_35378	Scarecrow-like transcription factor PAT1	33.560	6.59	60.22	6	0.774 ± 0.359	0.568 ± 0.018	0.813 ± 0.387
PEQU_12445	Cytochrome P450 71A1-like	31.560	8.1	57.79	2	1.018 ± 0.126	0.933 ± 0.006	0.921 ± 0.126
PEQU_41539	Allene oxide synthase 2-like	41.560	7.84	47.64	18	2.376 ± 0.991	3.853 ± 1.445	1.587 ± 0.103
PEQU_19521	FHA domain-containing protein DDL	20.560	10.86	44.49	1	1.194 ± 0.124	1.273 ± 0.263	1.050 ± 0.109
PEQU_02676	Oxygen-evolving enhancer protein 2, chloroplastic-like	54.560	8.95	28.5	9	1.446 ± 0.075	4.743 ± 0.124	3.368 ± 0.372
PEQU_02776	Oxygen-evolving enhancer protein 1, chloroplastic-like	55.560	7.89	35.73	17	1.217 ± 0.213	3.265 ± 0.424	2.783 ± 0.181
**Vernalization-related proteins**								
PEQU_11434	Glycine-rich RNA-binding protein GRP1A-like	24.560	6.86	13.65	5	1.741 ± 2.093	2.479 ± 1.676	2.969 ± 2.633
PEQU_11045	UDP-N-acetylglucosamine diphosphorylase 1-like	33.270	8.39	56.17	13	1.092 ± 0.156	1.71 ± 0.430	1.569 ± 0.194
PEQU_19304	Flowering locus T	22.560	7.51	19.75	7	2.240 ± 1.602	2.030 ± 0.649	1.062 ± 0.487
**Ubiquitin-related proteins**								
PEQU_03892	Auxin response factor 17-like	17.560	6.44	99.46	7	0.977 ± 0.366	1.683 ± 0.033	1.788 ± 0.594
PEQU_28956	Auxin response factor 17-like	28.560	8.37	22.66	7	0.895 ± 0.000	0.974 ± 0.063	1.078 ± 0.070
PEQU_26378	Ubiquitin-conjugating enzyme E2 36-like	29.560	7.51	17.18	8	0.836 ± 0.368	1.079 ± 0.538	1.283 ± 0.050
PEQU_21711	Ubiquitin-conjugating enzyme E2 7-like isoform X1	37.560	4.16	16.48	3	1.124 ± 0.380	1.113 ± 0.065	1.030 ± 0.291
PEQU_12056	Probable ubiquitin-conjugating enzyme E2 18	38.560	7.11	17.47	1	1.040 ± 0.095	1.082 ± 0.021	1.034 ± 0.074
PEQU_13455	NEDD8-conjugating enzyme Ubc12-like	39.560	8.31	20.85	2	0.997 ± 0.155	0.951 ± 0.031	0.958 ± 0.112
PEQU_22283	Cytochrome P450 90A1-like	2.679	6.94	51.26	1	0.943 ± 0.055	0.884 ± 0.052	0.825 ± 0.006
**Carbon- and energy metabolism-related proteins**								
PEQU_03049	Probable flavin-containing monooxygenase 1	19.560	7.59	61.7	2	0.829 ± 0.070	0.637 ± 0.025	0.763 ± 0.045
PEQU_21336	Glyceraldehyde-3-phosphate dehydrogenase A, chloroplastic	42.560	8.91	42.57	10	2.533 ± 0.459	11.914 ± 0.310	5.380 ± 1.821
PEQU_18900	LOW-QUALITY PROTEIN: ribulose bisphosphate carboxylase small chain clone 512-like	43.560	9.3	19.85	8	2.620 ± 1.336	12.538 ± 0.571	6.228 ± 2.635
PEQU_34221	Ferredoxin--NADP reductase, leaf-type isozyme, chloroplastic-like	44.560	8.72	40.83	10	0.963 ± 0.338	2.823 ± 0.439	3.110 ± 1.389
PEQU_02056	ATP synthase CF1 beta subunit (chloroplast)	45.560	5.25	53.5	27	1.225 ± 0.048	1.845 ± 0.012	1.507 ± 0.049
PEQU_01887	Ribulose bisphosphate carboxylase/oxygenase activase, chloroplastic-like isoform X2	46.560	7.36	51.78	15	2.071 ± 1.462	8.066 ± 1.256	5.560 ± 4.683
**Stress-related proteins**								
PEQU_18289	Beta-glucosidase 1-like	47.560	6.72	52.91	27	0.817 ± 0.608	0.368 ± 0.048	0.583 ± 0.373
PEQU_08910	GDP-mannose 3,5-epimerase 2	48.560	6.44	42.78	25	3.665 ± 0.095	3.158 ± 1.011	0.844 ± 0.260
PEQU_14594	Catalase isozyme A	49.560	7.06	56.35	28	3.370 ± 0.762	5.550 ± 1.325	1.652 ± 0.032
PEQU_05138	S-Adenosylmethionine synthase	50.560	6.74	37.76	12	1.905 ± 2.536	3.589 ± 1.389	6.971 ± 8.110
PEQU_15901	Alcohol dehydrogenase class-3	51.560	6.71	40.77	20	0.475 ± 0.316	0.199 ± 0.058	0.618 ± 0.567
PEQU_10731	Protein IN2-1 homolog B-like	52.560	7.58	47.15	18	2.813 ± 1.972	4.755 ± 1.955	1.876 ± 0.647

CK, flower buds cultivated at normal temperature for 0 d, 10 d, and 20 d; T10 group, flower buds under low-temperature treatment for 10 d; T20 group, flower buds under low-temperature treatment for 20 d.

**Table 2:** Proteins used for multi-reaction monitoring (MRM) verification.

Protein	GI Name	Fold Change of T10/CK	Adj. pvalue	Fold Change of T20/CK	Adj. pvalue	Fold Change of T20/T10	Adj. pvalue
PEQU_01887	Ribulose bisphosphate carboxylase/oxygenase activase, chloroplastic-like isoform X2	2.938	0.0001	23.137	<0.0001	7.876	<0.0001
PEQU_02056	ATP synthase CF1 beta subunit (chloroplast)	0.709	0.0023	1.344	0.0027	1.896	<0.0001
PEQU_02676	Oxygen-evolving enhancer protein 2, chloroplastic-like	0.966	0.4428	1.018	0.6831	1.054	0.2243
PEQU_02776	Oxygen-evolving enhancer protein 1, chloroplastic-like	0.801	0.0092	3.040	<0.0001	3.795	<0.0001
PEQU_03102	DEAD-box ATP-dependent RNA helicase 3, chloroplastic	2.445	0.0015	3.096	0.0002	1.266	0.1502
PEQU_07151	Probable methyltransferase PMT2	2.587	<0.0001	4.321	<0.0001	1.670	<0.0001
PEQU_08910	GDP-mannose 3,5-epimerase 2	2.247	<0.0001	3.929	<0.0001	1.749	0.0003
PEQU_10731	Protein IN2-1 homolog B-like	1.181	0.0218	3.182	<0.0001	2.693	<0.0001
PEQU_11434	Glycine-rich RNA-binding protein GRP1A-like	4.438	<0.0001	6.590	<0.0001	1.485	0.0010
PEQU_14594	Catalase isozyme A	1.508	0.0017	3.903	<0.0001	2.589	<0.0001
PEQU_15901	Alcohol dehydrogenase class-3	0.733	0.0079	0.479	<0.0001	0.654	0.0011
PEQU_16988	Homospermidine synthase	2.376	<0.0001	4.517	<0.0001	1.901	<0.0001
PEQU_18289	Beta-glucosidase 1-like	0.872	0.0260	0.350	<0.0001	0.402	<0.0001
PEQU_18900	LOW-QUALITY PROTEIN: ribulose bisphosphate carboxylase small chain clone 512-like	1.222	0.0346	3.444	<0.0001	2.818	<0.0001
PEQU_19304	Flowering locus T	2.438	<0.0001	2.586	<0.0001	1.061	0.1502
PEQU_21336	Glyceraldehyde-3-phosphate dehydrogenase A, chloroplastic	1.385	0.0150	10.707	<0.0001	7.731	<0.0001
PEQU_22307	Cryptochrome-1 isoform X1	0.775	0.0196	0.432	<0.0001	0.557	0.0003
PEQU_34221	Ferredoxin--NADP reductase, leaf-type isozyme, chloroplastic-like	1.057	0.4850	3.078	<0.0001	2.911	<0.0001

The ratios of proteins in the control and treatment groups and the adj.pvalue (adjusted p-value), which reflects false positives from the original statistical test used (method of Benjamini and Hochberg), are shown. Final target proteins with a fold change >1.5 and an adj.pvalue < 0.05 (false positive <0.05) were considered significantly differentially expressed proteins.

## References

[1] Ream T.S., Woods D.P., Amasino R.M. (2012). The molecular basis of vernalization in different plant groups. Cold Spring Harb. Symp..

[2] Bouché F., Woods D.P., Amasino R.M. (2017). Winter memory throughout the plant kingdom: Different paths to flowering. Plant Physiol..

[3] Brunner A.M., Evans L.M., Hsu C.Y., Sheng X. (2014). Vernalization and the chilling requirement to exit bud dormancy: Shared or separate regulation?. Front. Plant Sci..

[4] Komeda Y. (2004). Genetic regulation of time to flower in *Arabidopsis Thaliana*. Annu. Rev. Plant Biol..

[5] Smyth D.R., Bowman J.L., Meyerowitz E.M. (1990). Early flower development in *Arabidopsis*. Plant Cell.

[6] Xiao J., Xu S.J., Li C.H., Xu Y.Y., Xing L.J., Niu Y.D., Huan Q., Tang Y.M., Zhao C.P., Wagner D. (2014). O-GlcNAc-mediated interaction between VER2 and TaGRP2 elicits TaVRN1 mRNA accumulation during vernalization in winter wheat. Nat. Commun..

[7] Zhuang J.P., Huang S.Q., Pan S.Q., Ye Q.S. (2006). Construction and identification of a cDNA expression library from *Dendrobium nobile*. Acta Hortic. Sin..

[8] Huang J.Z., Lin C.P., Cheng T.C., Chang C.H., Cheng S.Y., Chen Y.W. (2015). A de novo floral transcriptome reveals clues into phalaenopsis orchid flower development. PLoS ONE.

[9] Chong K., Yong W.D., TAN K.H. (1999). Advances on research of vernalization in higher plants. Chin. Bull. Bot..

[10] Su W.R., Chen W.S., Koshioka M. (2001). Changes in gibberellin levels in the flowering shoot of *Phalaenopsis* hybrida under high temperature conditions when flower development is blocked. Plant Physiol. Biochem..

[11] Chen W.H., Hsu C.Y., Cheng H.Y. (2011). Downregulation of putative UDP-glucose: Flavonoid 3-O-glucosyltransferase gene alters flower coloring in *Phalaenopsis*. Plant Cell Rep..

[12] Suarez-Lopez P., Wheatley K., Robson F., Onouchi H., Valverde F., Coupland G. (2001). CONSTANS mediates between the circadian clock and the control of flowering in *Arabidopsis*. Nature.

[13] Ben-Naim O., Eshed R., Parnis A., Teper-Bamnolker P., Shalit A., Coupland G. (2006). The CCAAT binding factor can mediate interactions between CONSTANS-like proteins and DNA. Plant J..

[14] Valverde F., Mouradov A., Soppe W. (2004). Photoreceptor regulation of CONSTANS protein in photoperiodic flowering. Science.

[15] Fornara F., Panigrahi K.C.S., Gissot L. (2009). Arabidopsis DOF transcription factors act redundantly to reduce CONSTANS expression and are essential for a photoperiodic flowering response. Dev. Cell.

[16] Song Y.H., Estrada D.A., Johnson R.S. (2014). Distinct roles of FKF1, GIGANTEA, and ZEITLUPE proteins in the regulation of CONSTANS stability in *Arabidopsis* photoperiodic flowering. Proc. Natl. Acad. Sci. USA.

[17] Liu L.J., Zhang Y.C., Li Q.H. (2008). COP1-mediated ubiquitination of CONSTANS is implicated in cryptochrome regulation of flowering in *Arabidopsis*. Plant Cell.

[18] Song Y.H., Smith R.W., To B.J. (2012). FKF1 conveys timing information for CONSTANS stabilization in photoperiodic flowering. Science.

[19] Quesada V., Dean C., Simpson G.G. (2005). Regulated RNA processing in the control of *Arabidopsis* flowering. Int. J. Dev. Biol..

[20] Rodriguez-Cazorla E., Ripoll J.J., Andujar A., Bailey L.J., Martinez-Laborda A., Yanofsky M.F. (2015). K-homology nuclear ribonucleoproteins regulate floral organ identity and determinacy in *Arabidopsis*. PLoS Genet..

[21] Banerjee P., Hart G.W. (2014). O-linked N-acetylglucosamine (GlcNAc) transferase (UDP-N-acetylglucosamine: Polypeptide-N-acetylglucosaminyl transferase) (OGT). Handbook of Glycosyltransferases and Related Genes.

[22] Murase K., Hirano Y., Sun T.P., Hakoshima T. (2008). Gibberellin-induced DELLA recognition by the gibberellin receptor GID1. Nature.

[23] Daviere J.M., Achard P. (2013). Gibberellin signaling in plants. Development.

[24] Yu S., Galvao V.C., Zhang Y.C., Horrer D., Zhang T.Q., Hao Y.H. (2012). Gibberellin regulates the *Arabidopsis* floral transition through miR156-targeted SQUAMOSA promoter binding-like transcription factors. Plant Cell.

[25] Han Y., Chong K. (2004). Ubiquitin-proteasome pathway and regulation of auxin. Plant Physiol. Commun..

[26] Zhuang W., Gao Z., Wang L., Zhong W., Ni Z., Zhang Z. (2013). Comparative proteomic and transcriptomic approaches to address the active role of GA4 in Japanese apricot flower bud dormancy release. J. Exp. Bot..

[27] Chouard P. (1960). Vernalization and its relation to dormancy. Annu. Rev. Plant Physiol..

[28] Michaels S.D., Amasino R.M. (1999). FLOWERING LOCUS C encodes a novel MADS domain protein that acts as a repressor of flowering. Plant Cell.

[29] Danyluk J. (2003). TaVRT-1, a putative transcription factor associated with vegetative to reproductive transition in cereals. Plant Physiol..

[30] Trevaskis B., Bagnall D.J., Ellis M.H., Peacock W.J., Dennis E.S. (2003). MADS box genes control vernalization-induced flowering in cereals. Proc. Natl Acad. Sci. USA.

[31] Yan L. (2003). Positional cloning of the wheat vernalization gene VRN1. Proc. Natl Acad. Sci. USA.

[32] Distelfeld A., Li C., Dubcovsky J. (2009). Regulation of flowering in temperate cereals. Curr. Opin. Plant Biol..

[33] Song J., Angel A., Howard M., Dean C. (2012). Vernalization—A cold-induced epigenetic switch. J. Cell Sci..

[34] Greenup A., Peacock W.J., Dennis E.S., Trevaskis B. (2009). The molecular biology of seasonal flowering-responses in *Arabidopsis* and the cereals. Ann. Bot..

[35] Trevaskis B. (2010). The central role of the VERNALIZATION1 gene in the vernalization response of cereals. Funct. Plant Biol..

[36] Liang S., Ye Q.S., Li R.H., Leng J.Y., Li M.R., Wang X.J., Li H.Q. (2012). Transcriptional regulations on the low-temperature-induced floral transition in an orchidaceae species, dendrobium nobile: An expressed sequence tags analysis. Comp. Funct. Genom..

[37] Kim D.H., Doyle M.R., Sung S., Amasino R.M. (2009). Vernalization: Winter and the timing of flowering in plants. Annu. Rev. Cell Dev. Biol..

[38] Mangeon A., Junqueira R.M., Sachetto-Martins G. (2010). Functional diversity of the plant glycine-rich proteins superfamily. Plant Signal. Behav..

[39] Streitner C., Koster T., Simpson C.G., Shaw P., Danisman S., Brown J.W. (2012). An hnRNP-like RNA-binding protein affects alternative splicing by in vivo interaction with transcripts in *Arabidopsis thaliana*. Nucleic Acids Res..

[40] Zachara N.E., Hart G.W. (2004). O-GlcNAc a sensor of cellular state: The role of nucleocytoplasmic glycosylation in modulating cellular function in response to nutrition and stress. Biochim. Biophys. Acta.

[41] Yan L.L., Loukoianov A., Blechl A. (2004). The wheat VRN2 gene is a flowering repressor down-regulated by vernalization. Science.

[42] Xue W.Y., Xing Y.Z., Weng X.Y. (2008). Natural variation in Ghd7 is an important regulator of heading date and yield potential in rice. Nat. Genet..

[43] Osugi A., Itoh H., Ikeda-Kawakatsu K., Takano M., Izawa T. (2011). Molecular dissection of the roles of phytochrome in photoperiodic flowering in rice. Plant Physiol..

[44] Strasser B., Alvarez M.J., Califano A., Cerdan P.D. (2009). A complementary role for ELF3 and TFL1 in the regulation of flowering time by ambient temperature. Plant J..

[45] Yasui Y., Mukougawa K., Uemoto M., Yokofuji A., Suzuri R., Nishitani A. (2012). The phytochrome-interacting vascular plant one-zinc finger1 and VOZ2 redundantly regulate flowering in *Arabidopsis*. Plant Cell.

[46] Yasui Y., Kohchi T. (2014). VASCULAR PLANT ONE-ZINC FINGER1 and VOZ2 repress the FLOWERING LOCUS C clade members to control flowering time in *Arabidopsis*. Biosci. Biotechnol. Biochem..

[47] Yan L., Fu D., Li C. (2006). The wheat and barley vernalization gene VRN3 is an orthologue of FT. Proc. Natl. Acad. Sci. USA.

[48] Shitsukawa N., Ikari C., Shimada S. (2007). The einkorn wheat (Triticum monococcum) mutant, maintained vegetative phase, is caused by a deletion in the VRN1 gene. Genes Genet. Syst..

[49] Mertins P., Udeshi N.D., Clauser K.R. (2011). iTRAQ labeling is superior to mTRAQ for quantitative global proteomics and phosphoproteomics. Mol. Cell. Proteomics.

[50] Ow S.Y., Salim M., Noirel J. (2009). iTRAQ underestimation in simple and complex mixtures: “The good, the bad and the ugly”. J. Proteome Res..

[51] Huang S.Q., Guo J.J., Ye Q.S., Lin J.Y. (2003). Floral Induction and development in *Phalaenopsis* under different temperatures. Acta Scientiarum Naturalium Universitatis Sunyatseni.

[52] Yang S.H., Ji J., Wang G. (2008). Effect of nutrient formulation on growth of water culture *Phalaenopsis*. J. Agric. Univ. Hebei.

[53] Sun C.Q., Chen F.D., Fang W.M., Liu Z.L., Ma J., Teng N.J. (2009). Cellular mechanism of reproductive barrier during cross breeding between *Dendranthema grandiflorum* cv. Aoyuntianshi and *D. japonense*. Sci. Agric. Sin..

[54] Li M., Wang K., Li S.Q. (2016). Exploration of rice pistil responses during early post-pollination through a combined proteomic and transcriptomic analysis. J. Proteomics.

[55] Bradford M.M. (1976). A rapid and sensitive method for the quantitation of microgram quantities of protein utilizing the principle of protein-dye binding. Anal. Biochem..

[56] Cai J., Liu X., Vanneste K. (2015). The genome sequence of the orchid *Phalaenopsis equestris*. Nat. Genet..

[57] Martínez-Aguilar J., Molloy M.P. (2013). Label-free selected reaction monitoring enables multiplexed quantitation of S100 protein isoforms in cancer cells. J. Proteome Res..

[58] Tang H.Y., Beer L.A., Barnhart K.T., Speicheret D.W. (2011). Rapid verification of candidate serological biomarkers using gel-based, label-free multiple reaction monitoring. J. Proteome Res..

[59] Martins-de-Souza D., Alsaif1 M., Ernst A. (2012). The application of selective reaction monitoring confirms dysregulation of glycolysis in a preclinical model of schizophrenia. BMC Res. Notes.

[60] MacLean B., Tomazela D.M., Shulman N. (2010). Skyline: An open source document editor for creating and analyzing targeted proteomics experiments. Bioinformatics.

[61] Escher C., Reiter L., MacLean B. (2012). Using iRT, a normalized retention time for more targeted measurement of peptides. Proteomics.

